# Substantia nigra neuromelanin magnetic resonance imaging in patients with different subtypes of Parkinson disease

**DOI:** 10.1007/s00702-020-02295-8

**Published:** 2021-02-09

**Authors:** Lu Wang, Yayun Yan, Liyao Zhang, Yan Liu, Ruirui Luo, Ying Chang

**Affiliations:** Department of Neurology, China-Japan Union Hospital of Jilin University, Xiantai street 126, Changchun, 130033 Jilin Province China

**Keywords:** Neuromelanin, Magnetic resonance imaging, Parkinson disease, Motor subtypes

## Abstract

Neuromelanin (NM) is a dark pigment that mainly exists in neurons of the substantia nigra pars compacta (SNc). In Parkinson disease (PD) patients, NM concentration decreases gradually with degeneration and necrosis of dopamine neurons, suggesting potential use as a PD biomarker. We aimed to evaluate associations between NM concentration in in vivo SN and PD progression and different motor subtypes using NM magnetic resonance imaging (NM-MRI). Fifty-four patients with idiopathic PD were enrolled. Patients were divided into groups by subtypes with different clinical symptoms: tremor dominant (TD) group and postural instability and gait difficulty (PIGD) group. Fifteen healthy age-matched volunteers were enrolled as controls. All subjects underwent clinical assessment and NM-MRI examination. PD patients showed significantly decreased contrast-to-noise ratio (CNR) values in medial and lateral SN (*P* < 0.05) compared to controls. CNR values in lateral SN region decreased linearly with PD progression (*P* = 0.001). PIGD patients showed significant decreases in CNR mean values in lateral SN compared to TD patients (*P* = 0.004). Diagnostic accuracy of using lateral substantia nigra (SN) in TD and PIGD groups was 79% (sensitivity 76.5%, specificity 78.6%). NM concentration in PD patients decreases gradually during disease progression and differs significantly between PD subtypes. NM may be a reliable biomarker for PD severity and subtype identification.

## Introduction

Parkinson disease (PD) is a common neurodegenerative disorder, its pathological changes mainly focus on the dopaminergic neurons containing neuromelanin (NM) lost in the subatantia nigra pars compacta (SNc), and both noradrenergic and dopaminergic neurons containing NM lost in the locus coeruleus (Hirsch et al. 1988; Gibb 1992; Kastner et al.^1992^; Zarow et al. 2003). NM is a special dark polymer pigment, it mainly exist in the SNc and other catecholaminergic neurons. It has shown that NM is an important factor in promoting dopaminergic neurons degeneration in Parkinson disease (Hirsch et al. 1989; Kastner et al. 1992; Zecca et al. 2002). The mechanism may be that NM derived from dying neurons can activate microglia to release cytoactive factors, inducing the accumulation of the major histocompatibility complex class I (MHC-I), which can bind antigen and presenting on the membrane of the NM contained neurons, and then CD8 + T cells recognize and target these neurons, inducing neurodegeneration (Cerbrian et al. 2014). Pathological studies have shown that the degeneration of NM neurons occurs earlier than clinical symptoms appeared in PD patients, and it was exponentially lost with a 45% loss in the first decade of onset, and the number of NM in substantia nigra of PD patients after death is less than that 50% of the healthy control group (Fearnley and Lees 1991; Zecca et al. 2002). Therefore, NM may participate in the occurrence and development of PD through contribute to neuronal death, and become one of the pathogenic factors of PD. Sasaki et al. invented high-resolution spin-echo T1-weighted neuromelanin magnetic resonance imaging (NM-MRI), which revolutionized the visualization of neurons in the substantia nigra (Sasaki et al. 2006). Using high-resolution spin-echo T1-weighted NM magnetic resonance imaging (NM-MRI) can make the neurons with NM in the brain show high signal area to reflect the signal intensity and distribution of NM (Sulzer D et al. 2018). The loss of high signal area can be detected by NM-MRI in the early stage of Parkinson's disease. Therefore, we can clarify the progress of PD by noninvasive monitoring the attenuation degree of NM signal, and make NM become an in vivo biomarker reflecting the severity of PD (Cassidy et al. 2019).

Parkinson disease is a highly heterogeneous. Due to the lack of a unified classification standard, PD patients are often classified by clinical features into tremor dominant (TD) subtype and postural instability gait difficulty (PIGD) subtype (Stebbins et al. 2013). The clinical manifestations of the two subtypes were significantly different. Compared with patients with TD subtype, patients with PIGD subtype showed more severe symptoms, faster disease progression, higher depression tendency and more severe cognitive impairment (Konno et al. 2018; Dissanayaka et al. 2011). At present, the difference of neural mechanism between the two subtypes is not clear. The difference of NM concentration between different subtypes of PD detected by NM-MRI is helpful to deepen the understanding of the pathogenesis of these two subtypes and improve the efficacy of clinical intervention. Therefore, it is of great clinical significance for the treatment and prognosis of PD patients to strengthen the research on the neural mechanism of PD subtypes.

The purpose of this study was to explore the relationship between the concentration of NM and the progression of PD, and to explore the difference of NM signal intensity between the two subtypes of PD, so as to verify the feasibility of NM as a biomarker for identifying PD progression and subtypes.

## Subjects and methods

### Subjects and grouping

#### PD groups

A total of 54 patients with idiopathic PD were enrolled in this study. Inclusion criteria were: diagnosis by an experienced neurologist according to the Clinical Diagnostic Criteria for Parkinson disease developed by the International Parkinson and Movement Disorder Society (MDS) in 2015 (Postuma et al. 2015).

The exclusion criteria were: (1) secondary parkinsonism caused by repeated episodes of cerebrovascular disease, encephalitis, drugs, poisoning, infections, traumatic brain injury, etc.; (2) Parkinson plus syndromes such as multiple system atrophy, progressive supranuclear palsy, corticobasal degeneration, etc.; (3) those with chronic wasting diseases, such as severe liver and renal diseases, cardiopulmonary disease, endocrine disease or malignant tumor; (4)patients with a history of deep brain stimulation (DBS) surgery; (5) those with claustrophobia, pacemaker implantation, or poor compliance.

#### Scale scoring criteria

Disease severity was assessed using Parts II and III of the UPDRS scale and the H-Y stage when patients were in “off” periods (DaoKuan Liu 2000). In addition, the Mini-Mental State Examination (MMSE) was adopted to assess patients’ levels of cognitive function, and the Hamilton Depression Rating Scale (HAMD) was used to assess for possible depressive symptoms.

#### Motor typing standard

According to the grouping method developed by Stebbins et al. (2013), PD patients were divided into the TD group (*n* = 34), PIGD group (*n* = 14) and intermediate group (*n* = 6) according to the phenotype of their motor symptoms. The patients in the intermediate group were removed without further image acquisition and data analysis.

#### Control group

Additionally, this study enrolled a control group of 15 healthy age- and gender-matched volunteers who were in good health and had neither history of severe neurological/psychiatric disorders nor family history of neurological degeneration,and were evaluated by neurologists to rule out Parkinsonism.

### Ethical considerations

The Ethics Committee of our institution unanimously approved the protocol for this study, and all subjects provided signed informed consent to participate.

### Image acquisition

All NM-MRI images were obtained using a 3-T magnetic resonance imaging (MRI) scanner (Discovery™ MR750, GE Healthcare, Milwaukee, WI, USA). The patients were fully informed of precautions before the examination, and they were asked to lie on the examination table in a supine position while keeping quiet and calm during the scan. Foam pads were placed on both sides of the head to prevent head movement. A T1-weighted fast spin-echo sequence was used to acquire the images. The specific parameters were as follows: repetition time (TR)/echo time (TE) 500/11 ms; echo train length (ETL) 1; number of excitations (NEX) 8; slice thickness 2.0 mm; slice gap: 0.4 mm; number of slices acquired 20; matrix size 512 × 512; field of view 220 mm; and acquisition time 12 min. Twenty NM-MRI axial image slices parallel to the anterior–posterior commissure line of the corpus callosum were obtained for each individual. In addition, all subjects’ T1, T2, fluid attenuation inversion recovery (FLAIR), and diffusion-weighted images (DWI) were collected to exclude brain lesions that interfere with the experimental results. Note that the MRI images of PD patients were acquired in their relatively more energetic and mobile “on” state.

### Image processing

**Fig. 1:**
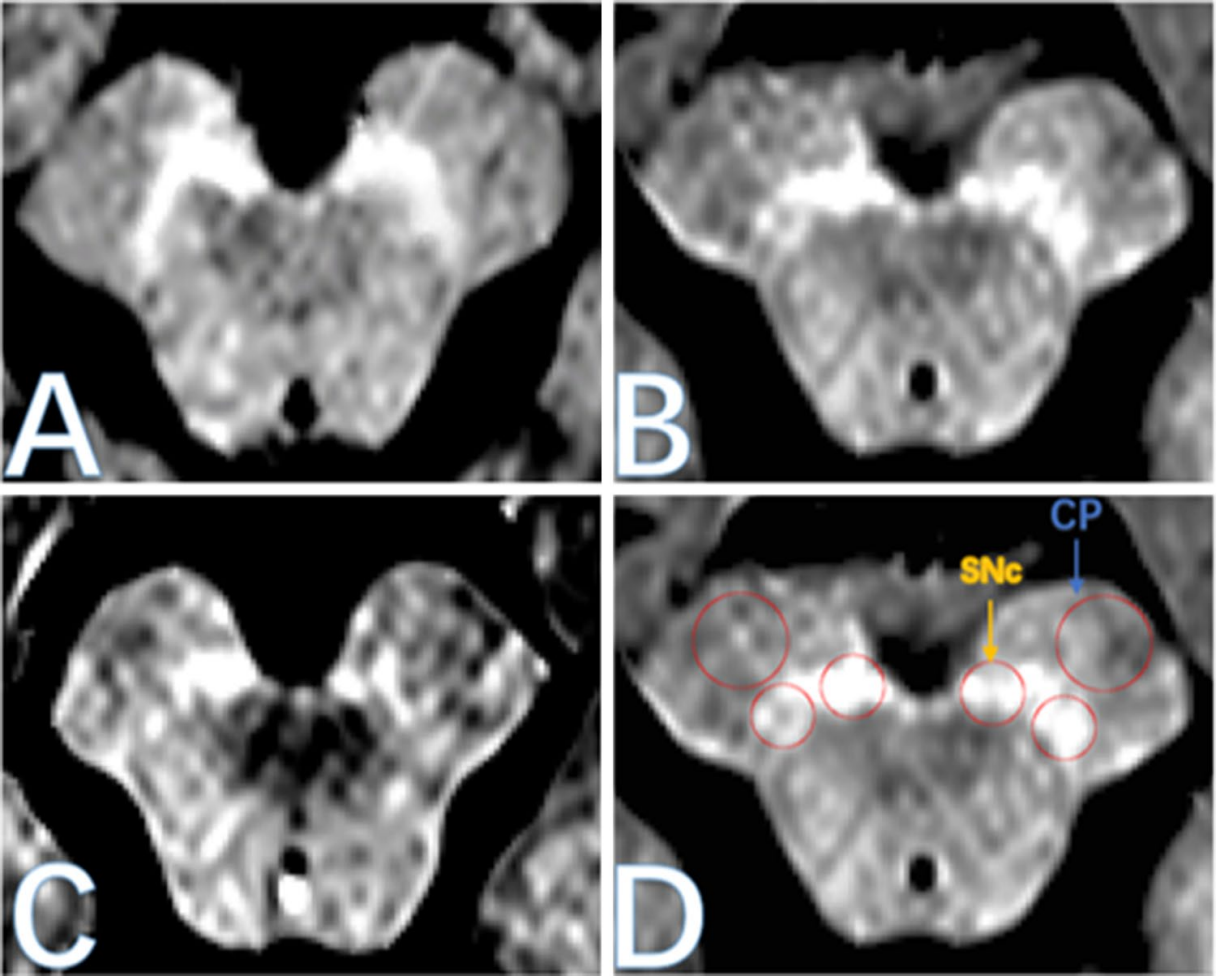
NM-MRI diagram of SN. **a** Healthy control group; **b** tremor dominant (TD); **c** group and postural instability and gait difficulty (PIGD) subgroup; **d** the circular region of interest for quantitative analysis is located in the lateral and medial parts of substantia nigra and left and right cerebral feet, *SN* substantia nigra pars, *CP* cerebral peduncles

Using image analysis software (Jim version 8.0, xinapse Systems, Colchester, United Kingdom; www.xinapse.com), three consecutive slices of high signal SN can be seen in the obtained axial image, the location and signal intensity distribution of the regions of interest (ROIs) were determined on the intermediate slice of SN according to the previous measurement methods (Schwarz et al. 2017; Reimão et al. 2015). Image analysis software (Jim Version 8.0, Xinapse Systems, Colchester, United Kingdom; www.xinapse.com) was used to determine the location and signal intensity distribution of the regions of interest (ROIs) on a slice containing SN (Konno et al. 2018). Then, the signal intensity of the ROIs, including the medial and lateral parts of the bilateral SN and cerebral peduncles (CP), were manually measured using the cursor on the display to obtain the average value after calculating the CNR values of the bilateral NM. Specifically, in the high-intensity medial and lateral parts of the bilateral SN, two circles with a radius of 2 mm were placed by a neurologist who was blinded to patient identity, and two circular ROIs with a radius of 3 mm were used as a control to determine the background signal of the CP (Fig. 1). The measurements were performed three times to compute the average value for subsequent statistical analysis. The intraclass correlation coefficient (ICC) of the measured values of ROIs is 0.83–0.91, which has good intra-observer consistency. Ultimately, the CNR of SN signal intensity was calculated using the following formula:

$${\text{CNR}} = \left( {{\text{SI}}_{{{\text{SN}}}} - {\text{SI}}_{{{\text{BG}}}} } \right)/{\text{SI}}_{{{\text{BG}}}} ,$$

where SI_SN_ is the signal intensity of the ROIs manually measured in the SN, and SI_BG_ is the background signal intensity of the ROIs in the bilateral CPs (Xing et al. 2018).

### Data analysis

Data of the TD, PIGD, and control groups were compared using analysis of variance (ANOVA) to identify statistically significant differences in age and gender between groups. The TD and control groups, and the TD and PIGD groups were compared separately using the rank-sum test to identify statistically significant differences in CNR, progression, MMSE and HAMD scores, H–Y stage, and UPDRS score between groups. Measurement data are expressed as median (quartile) [M (Q1, Q3)]. Partial correlation analysis was used to evaluate the statistical relationship between CNR measurements and course of disease of PD patients, and to correct for age and gender. Determine the intra-observer agreement by calculating the ICC value. All statistical tests were two-tailed, and *P* < 0.05 was considered to represent significant differences. The receiver operating characteristic curve (ROC curve) was adopted to determine the CNR values of the lateral SN in the TD and PIGD groups, so as to compare their sensitivity and specificity. The critical value was determined using the Jordan index. All statistical tests were performed using SPSS Statistics software (SPSS for Windows, version 23.0; SPSS Inc., Chicago, IL, USA) (Xing et al. 2018).

## Results

### Comparison of subjects’ demographic and clinical data

No significant differences were found in age (*P* = 0.782) and gender (*P* = 0.869) between the TD, PIGD, and control groups. Compared with the TD group, the PIGD group showed a significantly higher H–Y stage and UPDRSIII score (*P* = 0.008, 0.017), but no significant differences were found between groups in the course of disease, MMSE scores, MocA scores, and HAMD scores (*P* = 0.150, 0.437, 0.639 and 0.768, respectively, Table 1).

**Table 1:** General clinical data of subjects

	TD subgroup	PIGD subgroup	Control group	*P* value
Number of people (female/male)	34 (13/21)	14 (6/8)	15 (6/9)	0.869
Age (years)	63.5 (55.3, 70.8)	58 (54.3, 62.5)	61 (54.5, 69)	0.782
Duration of disease (years)	6 (4, 8.5)	7 (6.3, 9.8)	/	0.150
H–Y stage	2 (2, 2.5)	3 (2.1, 3)	/	0.008*
UPDRS part III score	49 (32.2, 64.5)	51 (82.5, 89.8)	/	0.017*
MMSE score	27 (25, 29)	27 (25.3, 28.8)	/	0.437
MocA scores	22.5 (19, 24.8)	24 (20, 24.8)	/	0.639
HAMD score	14.5 (11, 24)	17 (12.3, 18.8)	/	0.768

*TD* tremor dominant, *PIGD* group and postural instability and gait difficulty, *H–Y* Hoehn and Yahr, *UPDRS* unifified Parkinson disease rating scale, *MMSE* Mini-Mental State Examination, *HAMD* Hamilton Depression Rating Scale

*P < 0.05

### Comparison of CNR values in SN between PD and control groups

Compared with the control group, both PD groups showed decreased CNR values in the medial and lateral SN (*P* < 0.005, Table 2).

**Table 2:** Comparison of CNR value of substantia nigra between PD group and control group

	PD group	Control group	*P* value
Medial	0.14 (0.12, 0.16)	0.15 (0.14, 0.16)	0.049*
Lateral	0.09 (0.07, 0.10)	0.12 (0.12, 0.13)	< 0.001*

*CNR* contrast-to-noise ratio, *PD* Parkinson disease

*P < 0.05

### Associations between CNR values in the medial and lateral SN and PD course

**Fig. 2:**
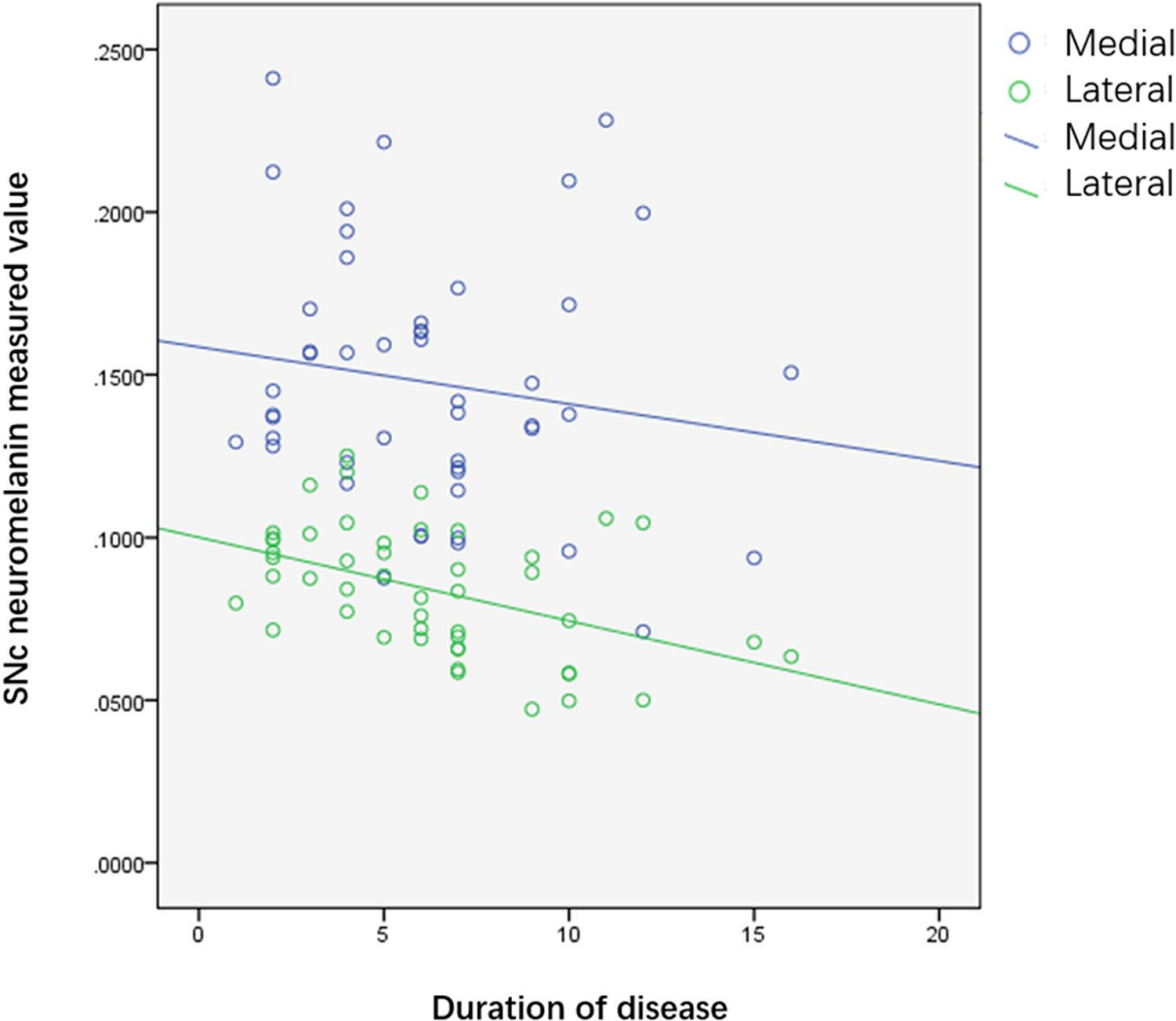
Scatter diagram of the relationship between CNR value of SN and duration of disease in PD patients. *CNR* contrast-to-noise ratio, *SN* substantia nigra pars, *PD* Parkinson disease

With the progression of PD, CNR values in the medial and lateral SN decreased linearly (*P* = 0.236, 0.008), particularly in the lateral part (Fig. 2).

### Comparison of CNR values in SN between the TD and PIGD groups

**Fig. 3:**
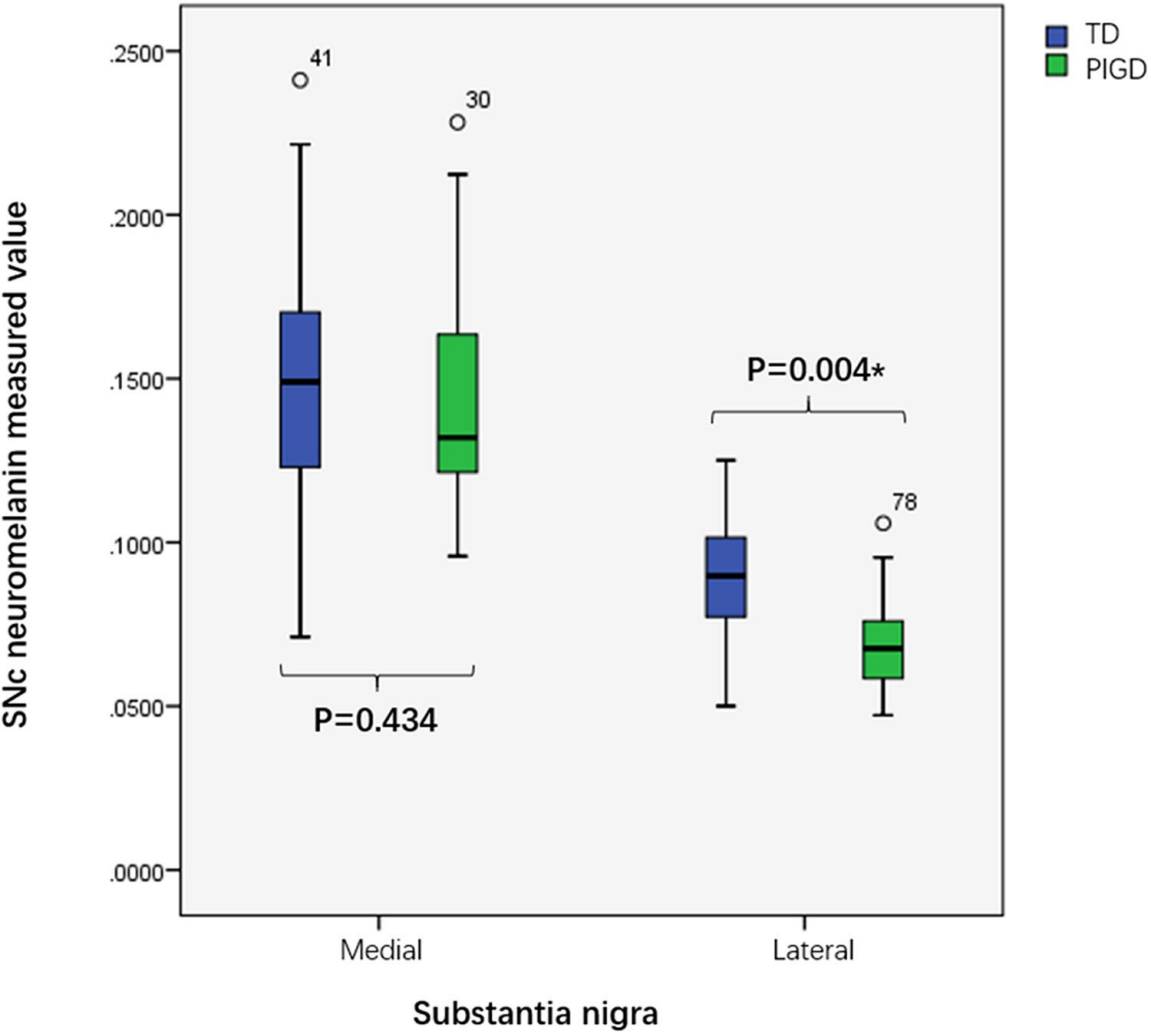
Box plot of CNR values of substantia nigra in TD and PIGD subgroups. *TD* tremor dominant, *PIGD* group and postural instability and gait difficulty, *CNR* contrast-to-noise ratio, *SN* substantia nigra pars. *P < 0.05

Compared with the TD group, the PIGD group had a significantly decreased CNR average value in the lateral SN (*P* = 0.004), but no statistically significant differences were found between the two groups in CNR values in the medial SN (*P* = 0.434, Fig. 3).

### Receiver operating characteristic (ROC) curve analysis of CNR values in the lateral SN between the TD and PIGD groups

**Fig. 4:**
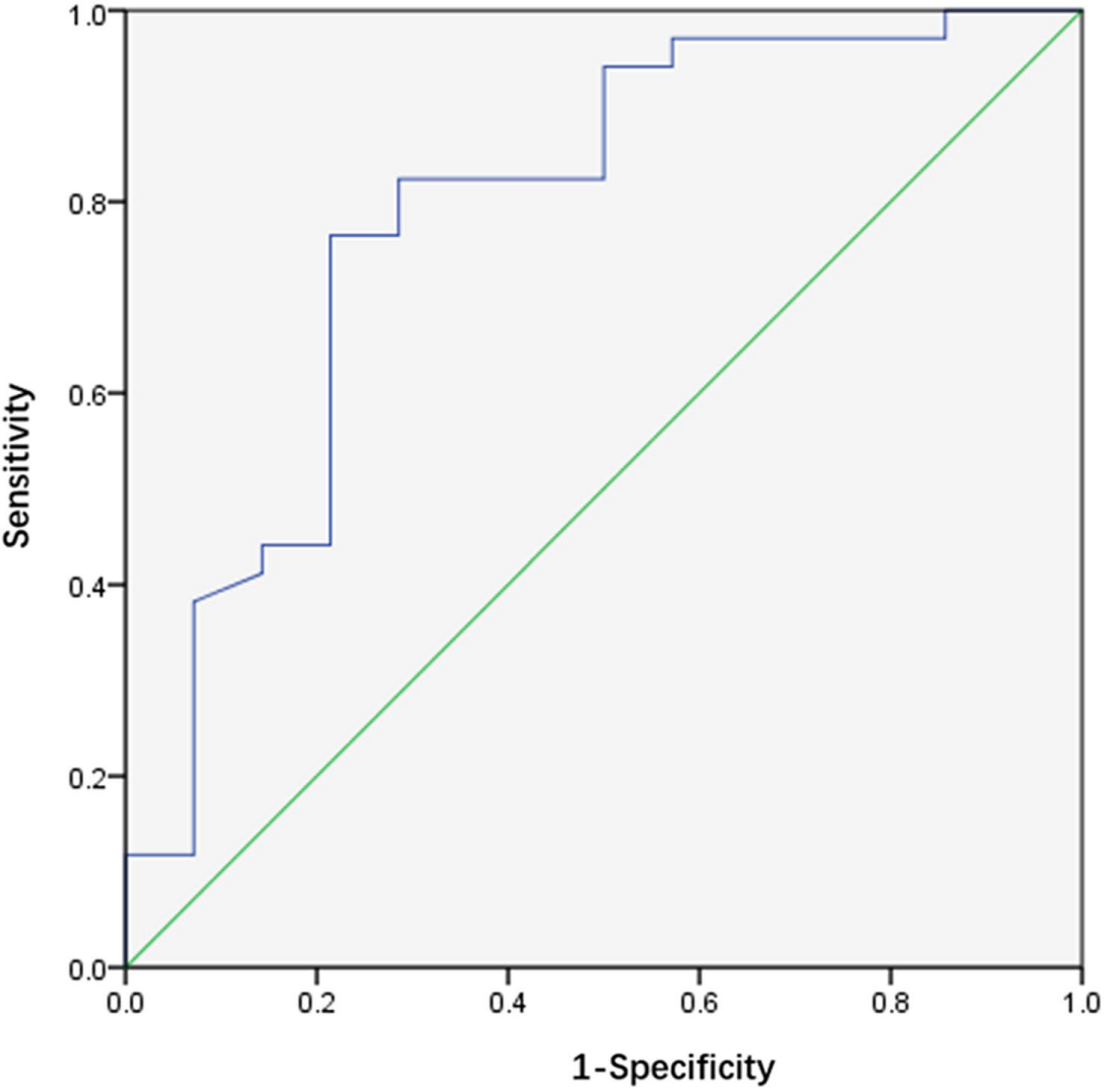
ROC curve of CNR value comparison of substantia nigra lateral part of TD and PIGD subgroups. *TD* tremor dominant, *PIGD* group and postural instability and gait difficulty, *CNR* contrast-to-noise ratio, *ROC* Receiver operating characteristic

The area under the ROC curve (AUC) of the lateral SN was 79% (sensitivity 76.5%, specificity 78.6%, and cut-off value 0.07), thereby demonstrating satisfactory diagnostic value (Fig. 4).

## Discussion

In the present study, we confirmed the correlation between NM and the progression of PD, and found that there was a significant difference in the degree of NM signal attenuation between the subtypes of PD patients: (1) CNR values were significantly decreased in PD patients in medial and lateral SN compared to controls, and decreased linearly with PD progression. (2) In PIGD patients, CNR mean values were significantly decreased compared to those in TD patients. H-Y stage and UPDRSIII scores were also significantly higher in the PIGD patients than those in the TD group.

PD is a progressive neurodegenerative disease whose characteristic pathological changes include progressive loss of NM-containing dopaminergic neurons and formation of Lewy bodies in the SN of the midbrain (Ehringer and Hornykiewicz 1960). The results of pathological anatomy showed that the loss rate of dopaminergic neurons in the SN of PD patients is proportional to the number of NM-containing cells therein, and that the loss rate of each NM-containing cell is positively associated with the amount of its melanin pigmentation. Therefore, there is a close clinicopathological correlation between NM and PD (Hirsch et al. 1988; 1989; Kastner et al. 1992; Zucca et al. 2018). In this study, NM-MRI results revealed that CNR values of NM were significantly lower than that of the healthy control group, which were consistent with previous research results (Reimão et al. 2015a, 2015b; Ohtsuka et al. 2013; Isaias et al. 2016). Meanwhile, we further analyzed the correlation between NM and the course of PD, and concluded that with the progression of PD, the clinical symptoms of patients were gradually aggravated, and the CNR values of medial and lateral SN decreased, especially in the lateral part. This result was consistent with the previous pathological research results. Previous pathological results showed that during the first 10 years of Parkinson's disease, the loss of pigmented neurons was exponential, with the largest loss in the ventrolateral layer (91%), followed by the ventrolateral layer (71%) and the dorsal lateral layer (56%) (Schwarz et al. 2017; Fearnley and Lees 1991). Most previous results of NM-MRI research also found that the decrease of NM-MRI signal in PD subjects was more inclined to the lateral, posterior and ventral voxels of SN, and NM is negatively associated with PD severity indicators, such as disease course, Hoehn–Yahr (H–Y) stage, and Unified Parkinson's Disease Rating Scale (UPDRS) motor score, but there are still a few studies showing no significant correlation (Cassidy et al. 2019; Schwarz et al. 2017; Reimão et al. 2015; Kashihara et al. 2011; Fabbri et al. 2017). This difference may be due to the application of different techniques in experimental data processing, which indicates that we still need to continue to explore the relationship between NM concentration and PD progression.

Our results also showed that there was a significant difference in signal attenuation in substantia nigra between pigd and TD groups. The signal attenuation of SN in the PIGD group was more serious than that in the TD group, which may be attributed to the difference of the two subtypes in pathophysiological mechanism. Autopsy results showed that compared with pigd patients, TD patients had less loss of pigment cells in SN, and less Lewy small volume aggregation in cortex (Selikhova et al. 2009). Therefore, it is speculated that the incidence of tremor is not closely associated with nigrostriatal pathway disorder, but instead it may result from the cerebellar–thalamic–cortical pathway. Therefore, the pigmented neurons containing NM in the substantia nigra of TD group had less degeneration and necrosis (Helmich et al. 2012). Our results showed that the signal attenuation of lateral SN in PIGD group was more serious than that in TD group (AUC 79%, sensitivity 76.5%, specificity 78.6%). There was no significant difference in CNR between the two groups. However, a previous small sample imaging study found that the signal attenuation difference between the two subgroups was more significant in the medial part of SN (Yuanyuan Xiang et al. 2017), while another NM-MRI study on de novo Parkinson disease showed no significant difference between the motor subgroups (Wang et al. 2018).

As the sample size of this study is relatively small, and the classification of PD motor subtypes may change with the progress of the disease (Eisinger et al. 2017), we need to follow-up the subjects for a longer time, and conduct a large sample longitudinal study to further clarify the difference of NM signal attenuation in substantia nigra between the two subgroups. Previous studies have shown that tremor is a feature of benign PD; compared with patients with bradykinesia and rigidity dominant PD, tremor dominant PD patients enter H–Y 4 more slowly (Rajput et al. 2009), have lower dementia and depression tendency (Vakil and Herishanu-Naaman 1998; Lewis et al. 2005; Williams-Gray et al. 2007; Aarsland et al. 2003; Williams-Gray et al. 2007; Van Rooden et al. 2011), Our results showed that the H–Y stage and updrsiii score of pigd group were significantly higher than those of TD group, but MMSE and hamd-24 scores had no significant difference, which could be attributed to the small number of Parkinson's disease patients registered in this study.

As a non-traumatic resting state brain imaging technology, NM-MRI does not require subjects to perform tedious tasks or receive invasive stimulation, provides favorable operability, and can be used as a routine clinical application to assist PD diagnosis and disease follow-up. Recent studies have shown that the volume and CNR of PD patients’ SN are positively associated with the dopamine transporter (DAT) density of its corresponding corpus striatum (Isaias et al. 2016). Therefore, NM-MRI can be used to quantitatively study the pathology of SN in PD patients, especially considering its close association with the dopaminergic innervation of the human striatum in PD.

## Limitations

This study has some limitations, including its relatively small sample size and cross-sectional study design. Considering the instability of the subtypes of some PD patients, it is necessary for the study to replicate in a larger sample. Another small sample study with 14 PD patients detected longitudinal changes in their SN values using NM-MRI. In that study, follow-up of the PD patients found them to have a significantly smaller total area of SN and a lower CNR value than the initial NM-MRI values (Matsuura et al. 2016). Therefore, it is feasible to consider NM-MRI as an indicator of PD progression. Furthermore, the motion artifacts during the acquisition of the NM-MRI sequence may have been generated by some TD patients and may have affected the sensitivity and specificity of the results. To confirm our results, additional prospective study with a larger sample and longitudinal exploration of the correlation between NM and PD severity is still needed.

## Conclusion

NM concentration in PD patients decreases gradually during disease progressionm with CMR values correlating linearly with the course of PD. In addition, PD patients in different subtypes demonstrate different rules of change for NM in the SN, NM may therefore be a reliable biomarker for PD severity and subtype identification. As such, we suggest that NM-MRI can be used for the identification of specific brain function changes during PD, thereby shedding new light on its pathological mechanism, diagnosis, and treatment.

## Data Availability

Date available.
